# *ApoE* gene polymorphisms and metals and their interactions with cognitive function

**DOI:** 10.1186/s12920-023-01632-6

**Published:** 2023-08-29

**Authors:** Zeyan Ye, Dechan Tan, Tingyu Luo, Ruoyu Gou, Jianshen Cai, Yanfei Wei, Kailian He, Song Xiao, Tingyu Mai, Xu Tang, Qiumei Liu, Xiaoting Mo, Yinxia Lin, Shenxiang Huang, You Li, Jian Qin, Zhiyong Zhang

**Affiliations:** 1 Department of Environmental Health and Occupational Medicine, School of Public Health, Guilin Medical University, The Guangxi Key Laboratory of Environmental Exposomics and Entire Lifecycle Heath, Zhiyuan Road No.1, Guilin, Guangxi province 541199 PR China; 2 Guangzhou Huashang Vocational College, No.1 Huashang Road, Lihu Street, Zengcheng District, Guangzhou, Guangdong Province 511300 China; 3 School of Public Health, Ningxia Medical University, Yinchuan, Ningxia 750004 China; 4 Department of Environmental and Occupational Health, Guangxi Medical University, Nanning, 530021 China; 5 Guangxi Health Commission Key Laboratory of Entire Lifecycle Health and Care, Guilin Medical University, Guilin, China

**Keywords:** Apolipoprotein E, *ApoE* gene polymorphism, Metallic elements, Cognitive function

## Abstract

**Objective:**

To analyze the relationship between plasma metal elements, *ApoE* gene polymorphisms and the interaction between the two and impaired cognitive function in elderly population.

**Method:**

A stratified sample was drawn according to the age of the study population, and 911 subjects were included. Baseline information and health indicators were obtained, and cognitive function status was assessed by health examination, a general questionnaire and Mini-Mental Status Examination. Plasma metal elements were measured, and SNP typing was performed. Binary logistic regression was used to analyze the factors influencing cognitive function status and the association between the SNP genetic pattern of the *ApoE* gene and cognitive function.

**Results:**

The differences in gene frequencies and genotype frequencies of the *ApoE* rs7412 and rs7259620 genotype frequencies were statistically different between the cognitive impairment group and the control group (*P* < 0.05). statistically differences were found for the codominant model in rs7412-TT compared with the CC genotype (OR = 3.112 (1.159–8.359), *P* = 0.024) and rs7259620-AA compared with the GG genotype (OR = 1.588 (1.007–2.504), *P* = 0.047). Statistically differences were found in the recessive models rs7412-TT compared with (CC + CT) (OR = 2.979 (1.112–7.978), *P* = 0.030), rs7259620-AA compared with (GG + GA), and rs405509-GG compared with (TT + TG) (OR = 1.548(1.022–2.344), *P* = 0.039) all of which increased the risk of developing cognitive impairment. The differences in plasma Fe, Cu, and Rb concentrations between the case and control groups were significant (*P* < 0.05). The regression results showed that the plasma Cd concentrations in the Q1 range was a protective factor for cognitive function compared with Q4 (0.510 (0.291–0.892), *P* = 0.018). Furthermore, there was a multiplicative interaction between the codominant and recessive models for the Q2 concentrations of Cd and the rs7259620 loci, and the difference was significant, indicating increased risk of developing cognitive impairment (codominant model OR = 3.577 (1.496–8.555), *P* = 0.004, recessive model OR = 3.505 (1.479–8.307), *P* = 0.004). There was also a multiplicative interaction between Cd and the recessive model at the rs405509 loci, and the difference was significant, indicating increased risk of developing cognitive impairment (OR = 3.169 (1.400-7.175), *P* = 0.006).

**Conclusion:**

The *ApoE* rs7412, rs7259620 and rs405509 loci were associated with cognitive impairment in the elderly population, and there was an interaction between plasma metalloid Cd and the rs7259620 and rs405509 loci that increased the risk of cognitive impairment in the elderly population.

## Introduction

In 2020, there were already 190 million people over 65 years old in China, accounting for 13.5% of China’s total population (National Bureau of Statistics of China). At the same time, population is aging rapidly in China, faster than those of many Asian, European and American countries [1, 2]. With the increasing aging of the population in contemporary society, the incidence of cognitive impairment (CI) is increasing yearly, especially in rural areas [3]. Studies have confirmed that cognitive impairment in the elderly population is mainly related to various factors, such as the environment, lifestyle, metal exposure, some genetic polymorphisms, and disease [4–6]. The etiology of cognitive impairment is still unclear, and the main mechanisms involve the gene mutation theory, the amyloid β-protein (Aβ) toxicity theory, the abnormal tau protein modification theory, and the oxidative stress theory [7]. There is no good way to prevent cognitive impairment.

Currently, 709 genes have been identified by genetic association analysis to be significantly associated with general cognitive function [8]. Among them, the gene encoding apolipoprotein E (*ApoE*) is located on chromosome 19 and has three isoforms, *ApoE*2, *ApoE*3 and *ApoE*4, which are expressed by alleles ɛ2, ɛ3 and ɛ4, respectively, *ApoE* may affect the metabolic deposition of amyloid β (Aβ) peptides, lipid metabolism, inflammatory response, and other mechanisms that cause cognitive impairment in the body through increased toxicity and/or loss of neuroprotective effects [9]. Moreover, different alleles of *ApoE* have different metal binding abilities [10]. The human body is exposed to a variety of metals in the natural environment at the same time, and metals enter the body through various pathways, such as the respiratory tract, digestive tract, and skin, and affect neurodevelopment through the immune, metabolic, and redox reactions of the human body. Excessive exposure to certain metals also has a certain effect on neurological function [11], The concentrations of metals such as cesium, manganese, barium, cadmium, lead, arsenic metabolites, and tungsten may be negatively correlated with cognitive function [12, 13]. There have been studies on the effects of different metal elements on cognitive function and indicate that metal dysregulation can cause cognitive deficits by inducing oxidative stress, synaptic damage and other pathways leading to disruption of neural networks [14], but no one has investigated the relationship between the interaction of *ApoE* with metal elements and cognitive function.

Guangxi Gongcheng Yao Autonomous County is dominated by the minority Yao population. The Yao have their unique customs and habits, such as drinking oil tea. Meanwhile, several studies on cognitive function in the population have been conducted in the area, and there is a relatively high prevalence of cognitive impairment in this area [15]; thus, it is useful to study the relationship between environmental factors and cognitive function. Based on the theory of the combined effect of genes and the environment, this study intends to assess the cognitive status of the population in Gongcheng Yao Autonomous County using the Mini-Mental State Examination (MMSE), analyze the association between plasma metal elements and *ApoE* gene polymorphisms and their interaction with cognitive function, then provide a theoretical basis for the prevention and treatment of cognitive dysfunction.

## Research subjects and methods

### Research subjects

The study population was patients undergoing health checkups of the Ecological Longevity Cross-sectional Study Project conducted in Gongcheng County, Guangxi, China, during December 2018 to December 2019. The inclusion criteria were as follows: (a) permanent residents of Gongcheng Yao Autonomous County, Guangxi Zhuang Autonomous Region; (b) people aged 65 years or older; (c) people who could cooperate to complete all health check-up items (including general examination, collection of blood and urine biological samples) and questionnaire surveys (including baseline and cognitive function-related questionnaires).

The exclusion criteria were as follows: (a) those with obvious visual and hearing impairment, language impairment, or physical disability; (b) those who could not cooperate to complete the health examination or questionnaire survey; (c) those with cognitive impairment due to organic encephalopathy, psychiatric diseases and other conditions; (d) those with missing or obvious logical errors in the key information of the physical examination results or questionnaire survey; (e) people with abnormal metal detection values (defined as 3 times the 99th percentile).

The target population size was determined according to the cross-sectional sampling formula $N=({Z}_{\alpha }^{2}\times pq)/{d}^{2}$. N is the sample size, Z is the statistic, α = 0.05, Z = 1.96, p is the expected prevalence, q = 1-p, and d is the permissible error, d = 0.1p. Using the 15.5% prevalence of cognitive impairment in the Chinese elderly population as a reference [16], the total sample size of the study was estimated to be 2095 people. The subjects were stratified according to their age, with a stratum for every 10 years, and each stratum was further divided into male and female. A total of 991 subjects were selected for plasma metal testing and SNP typing according to the overall proportion. The study protocol was approved by the medical ethics committee of Guilin Medical University (No: 20180702-3), and the subjects who participated in the study signed an informed consent form.

### Epidemiological investigation and biochemical testing

The investigators participating in this study had a medical background and were systematically trained before conducting the survey. The questionnaire was administered by means of face-to-face interviews with study participants using a standardized questionnaire under the principle of informed consent. The content of the questionnaire included the following aspects: basic information such as sex, age, ethnicity, education, occupation, and marital status. Lifestyle behaviors such as drinking, smoking, sleep and past history of disease.

The height, weight, and blood pressure of the subjects were measured. The range of normal blood pressure was 90–140 mmHg systolic and 60–90 mmHg diastolic; values outside this range were considered abnormal blood pressure. For all subjects, 16 ml of venous blood was taken from the elbow vein on an empty stomach after fasting for at least 12 h, divided into 4 blood collection tubes, kept refrigerated, and sent to the testing department of Gongcheng County People’s Hospital for health-related indices in a timely manner after the samples were collected on the same day. The plasma samples were centrifuged at 4 ℃ for 10 min and then tested for blood biochemical indices such as routine blood, glycosylated hemoglobin, lipids, and liver and kidney function. Plasma metal concentrations were determined by inductively coupled plasma-mass spectrometry (ICP-MS; Thermo Fisher scientific, USA) and other related content can be found in previous study [15].

### Cognitive function assessment

The Mini-Mental Status Examination was used to assess the cognitive status of the respondents [17]. The MMSE scale has a total score of 30 points and consists of 5 components: orientation, attention and calculation, memory, language, and visuospatial ability. Participants were grouped according to their MMSE scale scores and cultural education level and divided into cognitively normal and cognitively impaired groups as follows: the illiterate group with an MMSE score ≤ 16, elementary school group with an MMSE score ≤ 19, secondary school and above with an MMSE score ≤ 23 were considered to be in the cognitively impaired group, and the rest were in the cognitively normal group.

### Single-nucleotide polymorphisms and genotyping

#### SNP loci screening

The NCBI-SNP website was searched for functional SNP loci in the *ApoE* gene, promoter proxy (upstream variant 2 KB), 5’UTR, Exon (missense, synonymous), and 3’UTR regions; the loci obtained from the literature search were labeled and functional predictions were made for the screened loci.

#### Screening of validated and hot SNP loci

By searching the research literature on *ApoE* gene polymorphisms, SNP loci with susceptibility to the results were screened; candidate SNP loci were verified and functionally predicted according to the tools provided by the NIH website. The *ApoE* loci rs7259620, rs405509, rs429358, and rs7412 were finally screened, and the extracted DNA samples of the subjects were sent to Beijing Bo Miao Biotechnology Company. The MassARRAY flight mass spectrometry detection system was used for detection.

### Statistical analysis

EpiData 3.1 software was used to parallel double-entry questionnaires to establish the Guangxi Gongcheng Ecological Longevity Study cohort database. Statistical SPSS 26.0 software was used for analysis, Plink software for minimum allele frequency (MAF) analysis and Hardy-Weinberg equilibrium (HWE) law test. R 4.2.1 software package “Matrix”, “glmnet” and “foreign” were used for lasso regression, with parameters nlambda takes the default value of 100, and alpha is 1 for significant metal variable selection.

Continuous variables were expressed as the mean ± standard deviation (𝑥̅±s), The t test or Mann-Whitney U test was used for comparisons between groups; categorical variables were expressed as percentages (%), and the χ2 test was used for comparisons between groups. As the plasma metal assay results were right skewed, the plasma metal assay values were log_10_ transformed such that the data were approximately normally distributed for subsequent statistical analysis. After correction for confounders, dichotomous logistic regression was applied to analyze the factors affecting cognitive function. The interaction between genotypes of different genetic models of the *ApoE* gene rs429358, rs7412, rs7259620 and rs405509 at each of the four loci and plasma metal element levels on cognitive function was analyzed using a phase multiplication model. All statistical test levels were taken as α = 0.05.

## Results

### Characteristics of the study population

In the study population, the mean MMSE score was 16.00 ± 5.01 in the cognitively impaired group and 25.00 ± 3.42 in the cognitively normal functioning group, with a significant difference between the two groups (*P* < 0.05). A total of 991 subjects were included in this study, of whom 217 (21.9%) were in the cognitively impaired group and 774 (78.1%) were in the cognitively normal group. The mean age of the cognitively impaired group was 70.00 ± 6.69 years, and the mean age of the cognitively normal group was 69.00 ± 6.06 years. There were 63 males (29.03%) and 154 females (70.97%) in the cognitively impaired group and 386 males (49.87%) and 388 females (50.13%) in the cognitively normal group. The subjects were predominantly Yao (64.78%) and farmers (91.42%), the majority had spouses (71.75%), smoking (199, 20.08%), drinking (370, 37.34%), and had insomnia problems (543, 54.79%). A group comparison was made between the cognitive impairment group and the control group, and the differences in MMSE scores, sex, age, marital status, and diastolic blood pressure were significant between the two groups (*P* < 0.05). See Table 1.

**Table 1:** Comparison of general characteristics of study population n (%) ($\stackrel{-}{\varvec{x}}$±*s*)

Items	CI (N = 217)	Control (N = 774)	*χ*^*2*^/*t*	*p*
MMSE Score	16.00 ± 5.01	25.00 ± 3.42	23.567	0.000
Sex			25.332	0.000
male	63(29.03)	386(49.87)		
female	154(70.97)	388(50.13)		
Age	70.00 ± 6.69	69.00 ± 6.06	-1.992	0.047
Ethnicity			4.189	0.123
Han	59(27.19)	247(31.91)		
Yao	144(66.36)	498(63.34)		
Others	14(6.45)	29(4.75)		
marital status			5.454	0.020
No Spouse	75(34.56)	205(26.49)		
With Spouse	142(65.44)	569(73.51)		
occupation			1.601	0.206
Farmers	203(93.55)	703(90.83)		
Others	14(6.45)	71(9.17)		
Smoking			3.371	0.066
Yes	34(15.67)	165(21.32)		
No	183(84.33)	609(78.68)		
Drinking			0.103	0.748
Yes	79(36.41)	291(37.60)		
No	138(63.59)	483(62.40)		
Insomnia			0.829	0.362
Yes	113(52.07)	430(62.40)		
No	104(47.93)	344(37.6)		
BMI(kg/m^2^)	21.00 ± 3.39	22.00 ± 3.22	3.504	0.320
<18.5	34(15.67)	89(11.50))		
18.5–23.9	131(60.37)	469(60.56)		
24-27.9	45(20.74))	182(23.51)		
>27.9	7(3.22)	34(4.43)		
Systolic blood Pressure			0.430	0.512
Normal	105(48.39)	394(50.90)		
abnormality	112(51.61)	380(49.10)		
Diastolic blood pressure			5.236	0.022
Normal	133(61.29)	538(69.51)		
abnormality	84(38.71)	236(30.49)		
Gucose (mmol/L)	4.97 ± 1.70	4.96 ± 1.35	-0.872	0.383
LDL-C(mmol/L)	3.44 ± 0.93	3.55 ± 0.95	0.736	0.462
HDL-C(mmol/L)	1.87 ± 0.46	1.77 ± 0.45	0.628	0.111
TC(mmol/L)	4.97 ± 1.70	4.96 ± 1.35	-0.872	0.383
TG(mmol/L)	1.03 ± 1.01	1.03 ± 0.95	-0.204	0.839

### Basic information on SNP loci

The rs429358, rs7412, rs7259620, and rs405509 SNP loci of the *ApoE* gene were successfully typed, and the MAFs of each loci were greater than 0.05, indicating low-frequency variants. The equilibrium (HWE) law test (PHWE > 0.05), suggested a good representation of the selected population. See Table 2.

**Table 2:** Basic information on the SNPs

SNP	Chromosomes	Mutant/wild type allele	MAF	*P*_*HWE*_		
				Whole population	CI	Control
rs429358	19	C/T	0.089	0.846	1.000	0.827
rs7412	19	T/C	0.097	0.010	0.013	0.181
rs7259620	19	A/G	0.339	0.157	0.044	0.684
rs405509	19	G/T	0.345	0.035	0.014	0.334

### Allele frequency distribution of SNP loci

Comparison of the distribution of the alleles of the four SNP loci of the *ApoE* gene in the population and among the groups showed that rs7412 had statistically differences between the two groups (*P* < 0.05); meanwhile, the distribution of the alleles of the remaining three loci was similar, and none of the differences were significant. See Table 3.

**Table 3:** *ApoE* SNP allele frequency in the Study population n (%)

SNP	Allele	Total (N = 991)	Control (N = 774)	CI (N = 217)	*χ* ^*2*^	*p*
rs429358	T	1806(91.1)	1407(77.9)	399(22.1)		
	C	176(8.9)	141(80.1)	35(19.9)	0.337	0.562
rs7412	T	193(9.7)	137(71.0)	56(29.0)		
	C	1789(90.3)	1411(78.9)	378(21.1)	5.883	0.015
rs7259620	G	1310(66.1)	1040(79.4)	270(20.6)		
	A	672(33.9)	508(75.6)	164(24.4)	3.520	0.061
rs405509	T	1298(65.5)	1029(79.3)	269(20.7)		
	G	684(34.5)	519(75.9)	165(24.1)	2.830	0.093

### Genotype frequency distribution of SNP loci

The results of the genotype frequencies of the four SNP loci of the *ApoE* gene in the studied population and between groups showed that the genotype frequencies of rs7412 and rs7259620 were significantly different (*P* < 0.05) between the cognitive impairment and control groups, and the genotype distribution of the remaining loci was similar with no statistically differences. See Table 4.

**Table 4:** *ApoE* SNP genotype in the study population n (%)

SNP	Genotype	Total (N = 991)	Control (N = 774)	CI (N = 217)	*χ* ^*2*^	*p*
rs429358 T>C	TT	823(83.1)	640(77.8)	183(22.2)	0.620	0.734
	TC	160(16.1)	127(79.4)	33(20.6)		
	CC	8(0.8)	7(87.5)	1(12.5)		
rs7412 C>T	CC	815(83.2)	646(79.3)	169(20.7)	7.924	0.019
	CT	159(16.1)	119(74.8)	40(25.2)		
	TT	17(0.7)	9(52.9)	8(47.1)		
rs7259620 G>A	GG	443(44.7)	352(79.5)	91(20.5)	6.348	0.042
	GA	424(42.8)	336(79.2)	88(20.8)		
	AA	124(12.5)	86(69.4)	38(30.6)		
rs405509 T>G	TT	440(44.4)	348(79.1)	92(20.9)	5.676	0.059
	TG	418(42.2)	333(79.7)	85(20.3)		
	GG	133(13.4)	93(69.9)	40(30.1)		

### Association of genotype and allele frequency with cognitive impairment

Multifactorial logistic regression was used to analyze the association between SNP loci genotypes and four SNP inheritance patterns and cognitive impairment. In the codominant model, subjects carrying the TT allele of *ApoE* rs7412 had a 3.112 times higher risk of cognitive impairment than subjects with the CC genotype (OR = 3.112 (1.159–8.359), *P* = 0.024), and subjects carrying the AA allele of *ApoE* rs7259620 had a 1.588 times higher risk of cognitive impairment than subjects with the GG genotype (OR = 1.588 (1.007–2.504), *P* = 0.047).

In the recessive model, the risk of cognitive impairment was 2.979 times higher in subjects carrying the TT allele of *ApoE* rs7412 than in those with the (CC + CT) genotype (OR = 2.979 (1.112–7.978), *P* = 0.030), the risk of cognitive impairment was 1.558 times higher in subjects carrying the AA allele of *ApoE* rs7259620 than in those with the (GG + GA) genotype (OR = 1.558 (1.019–2.384), *P* = 0.041), and the risk of cognitive impairment was 1.548 times higher in subjects carrying the GG allele of *ApoE* rs405509 than in subjects with the (TT + TG) genotype (OR = 1.548 (1.022–2.344), *P* = 0.039), The correlation between genotype and cognitive functional status for the remaining SNP loci was not significant. See Table 5.

**Table 5:** Relationship between SNP gene pattern and cognitive impairment

SNP	Model	Genotype		*β*	Adjusted OR (confidence interval)	*P*
		Reference	Alternate			
rs429358 T>C	Co-dominant model	TT	TC	-0.054	0.947(0.618–1.451)	0.803
			CC	-0.582	0.559(0.067–4.652)	0.590
	dominant model	TT	TC + CC	-0.075	0.928(0.609–1.412)	0.726
	Recessive model	TT + TC	CC	-0.573	0.564(0.068–4.688)	0.596
	Over dominant model	TT + CC	TC	-0.050	0.952(0.621–1.457)	0.820
rs7412 C>T	Co-dominant model	CC	CT	0.253	1.288(0.859–1.933)	0.221
			TT	1.135	3.112(1.159–8.359)	0.024
	dominant model	CC	CT + TT	0.356	1.427(0.974–2.090)	0.068
	Recessive model	CC + CT	TT	1.092	2.979(1.112–7.978)	0.030
	Over dominant model	CC + TT	TC	0.222	1.249(0.834–1.872)	0.281
rs7259620 G>A	Co-dominant model	GG	GA	0.038	1.039(0.743–1.454)	0.823
			AA	0.462	1.588(1.007–2.504)	0.047
	dominant model	GG	GA + AA	0.147	1.158(0.849–1.581)	0.354
	Recessive model	GG + GA	AA	0.444	1.558(1.019–2.384)	0.041
	Over dominant model	GG + AA	GA	-0.076	0.927(0.678–1.267)	0.635
rs405509 T>G	Co-dominant model	TT	TG	0.002	1.002(0.715–1.406)	0.989
			GG	0.438	1.550(0.992–2.420)	0.054
	dominant model	TT	TG + GG	0.121	1.129(0.827–1.541)	0.444
	Recessive model	TT + TG	GG	0.437	1.548(1.022–2.344)	0.039
	Over dominant model	TT + GG	TG	-0.110	0.895(0.654–1.226)	0.491

*P* values were adjusted for sex, age, marital status, and dichotomous diastolic blood pressure

### Distribution of plasma metal element concentrations

The plasma metal concentrations were skewed, and the assay values were statistically analyzed after log_10_ transformation. The plasma metal concentrations of Mg, Cr, Mn, Cu, As, Sr, Mo, Cd, Sn, Sb, and Pb were higher in the cognitively impaired group than in the cognitively normal group. Additionally, the results of the comparison between groups showed that the differences between the two groups in Fe, Cu, and Rb were significant (*P* < 0.05) in the cognitively impaired group and the normal group. See Table 6.

**Table 6:** Distribution of 22 plasma metals in the plasma of the study population

Metal	Total population (ug/L)	CI group (ug/L)	Control group (ug/L)	*t*	*P*
Mg	17817.76(16482.17-19519.92)	17924.69(16213.47-19768.82)	17812.08(16569.52-19424.78)	0.04	0.968
Al	66.67(28.60-111.21)	65.25(28.07-107.33)	66.86(28.96-111.74)	-0.166	0.858
Ca	69585.4232(65676.37-74059.27)	69192.21(64911.50-74517.25)	69791.34(65747.57-73944.22)	0.702	0.483
Ti	16.75(14.34–20.64)	16.49(13.93–20.31)	16.82(14.51–20.67)	-0.364	0.716
V	0.27(0.19–0.52)	0.26(0.18–0.52)	0.27(0.19–0.52)	-1.263	0.072
Cr	2.46(1.58–3.84)	2.57(1.44–3.94)	2.39(1.54–3.81)	-1.263	0.207
Mn	1.81(1.41–2.72)	1.87(1.44–2.78)	1.80(1.41–2.71)	-0.828	0.408
Fe	1.67.37(830.44-1342.04)	972.77(736.41-1216.47)	1099.59(849.80-1364.46)	3.149	0.002
Ni	4.88(4.02-6.00)	4.75(3.87–5.78)	4.94(4.04–6.05)	1.170	0.242
Co	0.21(0.14–0.54)	0.21(0.14–0.50)	0.21(0.14–0.56)	0.068	0.946
Cu	939.64(818.36-1066.67)	957.60(859.73-1075.48)	934.12(808.84-1061.65)	-2.324	0.021
Zn	937.43(718.71-1799.52)	935.90(710.23-1871.60)	939.27(723.87-1781.93)	-0.846	0.398
As	1.17(0.94–1.77)	1.21(0.95–2.10)	1.17(0.94–1.73)	-1.279	0.201
Se	109.29(93.22–126.50)	105.53(90.47-125.58)	110.01(94.22-127.24)	1.654	0.099
Rb	410.53(345.99-501.55)	392.74(336.34-473.38)	417.23(347.81-507.51)	2.400	0.017
Sr	25.17(19.74–31.32)	25.78(19.99–31.10)	25.00(19.55–31.48)	-0.843	0.399
Mo	1.74(1.33–2.28)	1.84(1.38–2.44)	1.73(1.31–2.24)	0.174	0.862
Cd	0.19(0.12–0.30)	0.19(0.13–0.30)	0.18(0.12–0.29)	-1.751	0.080
Sn	1.22(0.66–2.25)	1.38(0.70–2.37)	1.14(0.66–2.23)	-0.789	0.430
Sb	1.37(0.60–5.10)	1.38(0.71–4.95)	1.36(0.58–5.12)	0.607	0.544
Ba	21.62(15.84–29.55)	20.73(15.02–29.31)	21.93(15.99–29.58)	1.326	0.185
Pb	4.47(3.12–7.83)	4.76(2.96–8.49)	4.46(3.15–7.46)	-0.275	0.783

### Selection of metal elements

**Fig. 1:**
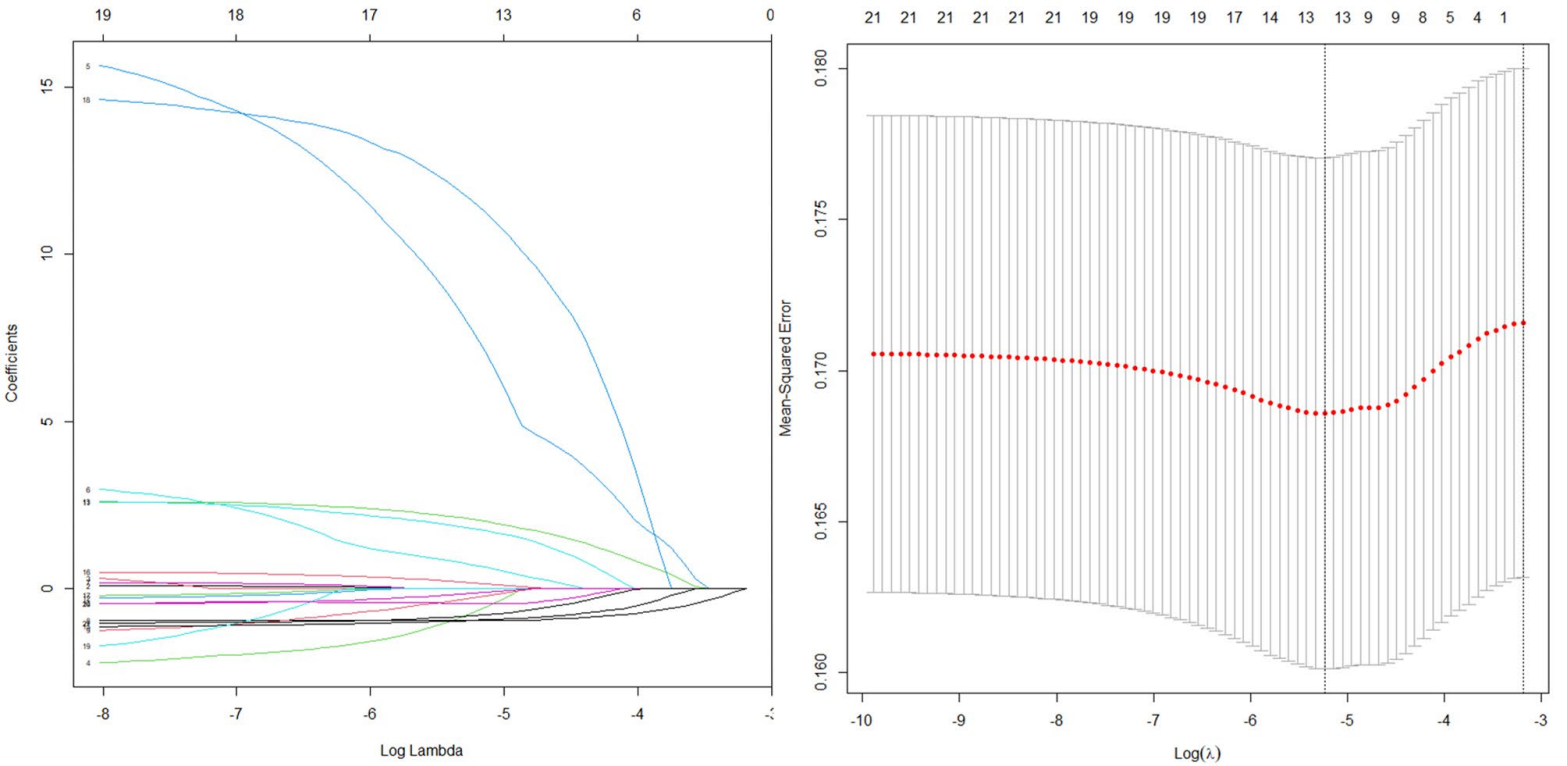
Changes in the LASSO regression coefficient and lambda and cross-validation results

The important characteristic metal elements associated with cognitive impairment were screened out from 22 metals by LASSO regression, and thirteen metal elements, Ti, V, Cr, Fe, Ni, Cu, As, Se, Rb, Sr, Cd, Sb, and Ba, were ultimately screened out and included in the subsequent analysis. See Fig. 1.

### Association of plasma metal elements with cognitive function

The results showed a positive correlation between elemental Cd concentration and cognitive impairment, with the risk of cognitive impairment being 0.510 times higher in the Q1 concentration range population than in the Q4 (OR = 0.510 (0.291–0.892)), *P* = 0.018), and the differences for the remaining metal were not significant. See Table 7.

**Table 7:** Logistic regression of the relationship between plasma metal and cognitive function

Matal	*β*	OR (*95%CI*)	*P*
Constants	-3.345		
Ti			0.326
Q_1_	0.227	1.254(0.628–2.504)	0.521
Q_2_	-0.189	0.827(0.421–1.627)	0.583
Q_3_	-0.119	0.888(0.479–1.647)	0.707
Q_4_		1	
V			0.062
Q_1_	0.663	1.940(0.904–4.162)	0.089
Q_2_	0.068	1.070(0.546–2.099)	0.843
Q_3_	-0.040	0.961(0.506–1.826)	0.903
Q_4_		1	
Cr			0.409
Q_1_	-0.458	0.633(0.339–1.181)	0.150
Q_2_	-0.084	0.919(0.529–1.597)	0.765
Q_3_	-0.057	0.944(0.591–1.509)	0.810
Q_4_		1	
Fe			0.077
Q_1_	0.481	1.618(0.914–2.862)	0.098
Q_2_	0.268	1.308(0.778–2.199)	0.312
Q_3_	-0.184	0.832(0.490–1.413)	0.496
Q_4_		1	
Ni			0.476
Q_1_	-0.220	0.802(0.428–1.502)	0.491
Q_2_	0.144	1.155(0.683–1.954)	0.590
Q_3_	-0.119	0.888(0.529–1.490)	0.652
Q_4_		1	
Cu			0.062
Q_1_	-0.270	0.764(0.460–1.267)	0.297
Q_2_	0.365	1.441(0.916–2.267)	0.114
Q_3_	0.222	1.249(0.795–1.960)	0.334
Q_4_		1	
As			0.668
Q_1_	-0.175	0.839(0.510–1.381)	0.491
Q_2_	-0.282	0.754(0.466–1.222)	0.252
Q_3_	-0.241	0.786(0.492–1.256)	0.314
Q_4_		1	
Se			0.595
Q_1_	0.270	1.310(0.773–2.220)	0.316
Q_2_	-0.029	0.971(0.590–1.599)	0.908
Q_3_	-0.002	0.998(0.612–1.626)	0.992
Q_4_		1	
Rb			0.226
Q_1_	0.191	1.211(0.723–2.028)	0.467
Q_2_	0.433	1.542(0.949–2.503)	0.080
Q_3_	0.004	1.004(0.613–1.647)	0.986
Q_4_		1	
Sr			0.409
Q_1_	-0.240	0.787(0.469–1.320)	0.364
Q_2_	-0.232	0.793(0.486–1.293)	0.353
Q_3_	0.096	1.101(0.693–1.750)	0.683
Q_4_		1	
Cd			0.084
Q_1_	-0.674	0.510(0.291–0.892)	0.018
Q_2_	-0.142	0.868(0.538–1.399)	0.561
Q_3_	-0.088	0.916(0.573–1.463)	0.713
Q_4_		1	
Sb			0.271
Q_1_	-0.193	0.824(0.463–1.466)	0.510
Q_2_	0.298	1.347(0.808–2.245)	0.253
Q_3_	0.096	1.101(0.658–1.843)	0.714
Q_4_		1	
Ba			0.243
Q_1_	0.411	1.508(0.880–2.584)	0.135
Q_2_	0.106	1.112(0.671–1.844)	0.680
Q_3_	-0.085	0.918(0.554–1.521)	0.740
Q_4_		1	

The above 12 metal elements above were included in a binary logistic regression analysis, while the study population was quadratically transformed (Q1, Q2, Q3, and Q4) according to the concentrations of the above metal elements in plasma with those with plasma metal concentrations ranging from Q4 as controls. *p* values were adjusted for sex, age, marital status and dichotomous diastolic blood pressure.

### Association analysis of *ApoE* gene and plasma metal interactions with cognitive function

The results of the interaction of the metal element Cd showed that the interaction group of the rs7259620-AA genotype with the Q2 concentration of Cd in the codominant model had a significantly higher risk of developing cognitive impairment than the interaction group of the rs7259620-GG genotype with the Q4 concentration (OR = 3.577 (1.496–8.555), *P* = 0.004). The risk of cognitive impairment was significantly higher in the recessive model in the rs7259620-AA genotype interacting with Q2 concentrations of Cd compared with the rs7259620-(GA + GG) genotype interacting with Q4 concentrations (OR = 3.505 (1.479–8.307) *P* = 0.004), The rs405509-GG genotype interacted with Q2 concentrations of Cd compared with the rs405509-(TT + TG) genotype, which interacted with Q4 concentrations (OR = 3.169 (1.400-7.175), *P* = 0.006). See Table 8.

**Table 8:** Logistic regression analysis of the interaction between SNP sites and metals

SNP	Interaction	OR (confidence interval)	*P*-value
Cd *SNPs			
Q_1*_rs7412	Co-dominant model TT	0.539(0.062–4.671)	0.575
	Recessive model TT	0.517(0.060–4.471)	0.549
Q_2*_rs7412	Co-dominant model TT	3.511(0.217–56.916)	0.377
	Recessive model TT	3.365(0.208–54.517)	0.393
Q_3*_rs7412	Co-dominant model TT	3.855(0.727–20.446)	0.113
	Recessive model TT	3.691(0.697–19.559)	0.125
Q_1*_rs7259620	Co-dominant model AA	1.027(0.463–2.275)	0.948
	Recessive model AA	1.006(0.458–2.206)	0.989
Q_2*_rs7259620	Co-dominant model AA	3.577(1.496–8.555)	0.004
	Recessive model AA	3.505(1.479–8.307)	0.004
Q_3*_rs7259620	Co-dominant model AA	1.834(0.857–3.925)	0.118
	Recessive model AA	1.797(0.849–3.803)	0.126
Q_1*_rs405509	Recessive model GG	1.021(0.467–2.230)	0.959
Q_2*_rs405509	Recessive model GG	3.169(1.400-7.175)	0.006
Q_3*_rs405509	Recessive model GG	1.805(0.881–3.697)	0.107

## Discussion

This study included 991 participants. There were 217 people in the cognitive impairment group, with a cognitive impairment rate of 21.9%, which is higher than the prevalence of mild cognitive impairment of 15.5% among Chinese elderly people [16], and the 10.8% prevalence of cognitive impairment in the Canadian older adult population reported by Chihireh B et al. [18], but lower than the prevalence of 23.4% reported by Unverzagt FW et al. in an elderly African American population [19]. The differences may be due to the different methods currently used to assess cognitive function status, inconsistent criteria for determining cognitive impairment, the slightly different incidence of cognitive impairment in different regions, or the differences due to genetic characteristics, local natural environment, dietary characteristics, and gene-environment interactions in different study populations in different regions.

Our study found that sex, age, marital status, and diastolic blood pressure were influential factors for cognitive impairment. The higher risk of cognitive impairment in women than in men is well established [20], and age is a well-recognized factor influencing cognitive functional status. With increasing age, the risk of cognitive impairment gradually increases as the body organs and functions gradually decline, the functional brain structure atrophies and cognitive function also gradually declines. Additionally, the proportion of spouselessness in the cognitive impairment group was higher than that in the control group, which is the same as the findings of LIU H et al. [21]; meanwhile, Chen ZC et al. noted that long-term spouselessness was associated with a higher risk of developing cognitive impairment than short-term spouselessness [22]. The difference in diastolic blood pressure between the cognitive impairment group and the normal group was the same as that obtained by Yanjun Ma et al. for three representative aging cohorts [23]. A meta-analysis of the results of a randomized clinical trial concluded that lowering blood pressure with antihypertensive medication significantly reduce the risk of developing cognitive impairment in the population compared with the control group [24].

The results of the analysis of 22 plasma metal elements after correction for confounding factors showed that Cd may be an influential factor in decreasing cognitive function. The mean plasma Cd concentration of 0.19 µg/L in the elderly population in this study was lower than the mean plasma Cd concentration of 0.50 µg/L in the U.S. elderly population aged 60–80 years included in the study by Li et al. [25]. This may be due to differences such as the regional diet of the study population. In our study, Cd showed a positive correlation with cognitive impairment compared with the Q4 concentration range, which is in line with some of the current studies [26, 27]. Cd accumulation can affect the integrity and permeability of the vascular endothelium and cross the blood-brain barrier with the assistance of carriers such as ethanol, which can lead to neuronal necrosis and affect cognitive function [28, 29]. Additionally, dysregulated metals in vivo can lead to impaired cognitive function through oxidative stress pathways [30].

The results of the association analysis between genes and cognitive function showed that the rs7412 and rs7259620 alleles of the *ApoE* gene in codominant and recessive models and the rs405509 allele in the recessive model were associated with cognitive functional status, consistent with the findings of Talwar et al. [31]. The rs429358 allele in this study did not differ significantly between the case and control groups, consistent with the findings of a study of a small population with schizophrenia [32], but contrary to the findings of Zhen et al., who concluded that the *ApoE* gene rs429358 was significantly associated with cognitive function in a middle-aged and older male population [33]. The *ApoE* gene has specific relevance to memory function in the central nervous system, and its expression regulation and gene polymorphisms are closely related to neurological or neurodegenerative diseases such as Alzheimer’s disease, Parkinson’s disease, and hyperlipidemia [34–36]. The discrepancy between findings may be due to the different populations studied.

In the interaction model with metals, different alleles and loci of *ApoE* have different abilities to bind to metals. In this study, we found that the codominant and recessive models of the *ApoE* rs7259620 loci with the AA genotype and the recessive model of the rs405509 loci with the GG genotype interacted with the Q2 concentration range of Cd in a multiplicative manner compared with the Q4 concentration range, and their interaction with the rs7259620 and rs405509 loci would change from the protective effect of low concentration of Cd to a risk factor. It has been suggested that rs7259620 is associated with neuropathic features [37] and that the AA genotype in the *ApoE* gene may be an independent risk factor for cognitive impairment compared with other genotypes [38]. Lambert et al. found differences between alleles G and T when providing evidence for other mutation hypotheses near the *ApoE* gene [39], while a study by Ding N et al. noted that the *ApoE* gene may be influenced by metals and exhibit susceptibility to disease development [40]. These results suggest that the rs7259620-AA and rs405509-GG genotypes may interact with the Q2 concentration range of Cd to increase the risk of cognitive dysfunction.

## Conclusion

A study of baseline data for this population found that differences in age, gender, marital status, and diastolic blood pressure affected cognitive function in the population, while plasma metal Fe, Cu, and Rb levels differed between the cognitively impaired and control groups. The *ApoE* rs7412 allele frequency and rs7412 and rs7259620 genotype frequencies were statistically different between the cognitively impaired and control groups. In the co-dominant and recessive models, those carrying rs7412 allele TT and rs7259620 allele AA will likely increase their risk of cognitive impairment. In the recessive model carrying rs405509 allele GG may increase the risk of cognitive impairment. And the presence of plasma metal element Cd interacting with rs7259620 and rs405509 loci in the elderly population will increase the risk of cognitive impairment.

## Limitations

The present study has some limitations. First, the study was a cross-sectional study, and inferring the relationship between genes and cognition, environmental factors and cognition, and gene-gene and gene-environment interactions and cognition is not yet sufficient and still has some limitations; furthermore, follow-up of the subjects is pending. Second, although we adjusted for some potential confounders, we could still not avoid the possibility of residual confounders, and this association must be further investigated by prospective studies with large samples.

## Data Availability

The datasets used and/or analysed during the current study available from the corresponding author on reasonable request.
